# Neuroimaging studies of alexithymia: physical, affective, and social perspectives

**DOI:** 10.1186/1751-0759-7-8

**Published:** 2013-03-28

**Authors:** Yoshiya Moriguchi, Gen Komaki

**Affiliations:** 1 Department of Psychophysiology, National Center of Neurology and Psychiatry, 4-1-1 Ogawahigashi, Kodaira, Tokyo, 187-0031, Japan; 2 Integrative Brain Imaging Center, National Center of Neurology and Psychiatry4-1-1 Ogawahigashi, Kodaira, Tokyo, 187-0031, Japan; 3 Fukuoka School of Rehabilitation Sciences, International University of Health and Welfare, 137-1, Enokizu, Ohkawa, Fukuoka, 831-0004, Japan

**Keywords:** Alexithymia, fMRI, PET, Neuroimaging study, Interoception, Psychosomatic medicine, Emotional awareness

## Abstract

Alexithymia refers to difficulty in identifying and expressing one’s emotions, and it is related to disturbed emotional regulation. It was originally proposed as a personality trait that plays a central role in psychosomatic diseases. This review of neuroimaging studies on alexithymia suggests that alexithymia is associated with reduced neural responses to emotional stimuli from the external environment, as well as with reduced activity during imagery, in the limbic and paralimbic areas (i.e., amygdala, insula, anterior/posterior cingulate cortex). In contrast, alexithymia is also known to be associated with enhanced neural activity in somatosensory and sensorimotor regions, including the insula. Moreover, neural activity in the medial, prefrontal, and insula cortex was lowered when people with alexithymia were involved in social tasks. Because most neuroimaging studies have been based on sampling by self-reported questionnaires, the contrasted features of neural activities in response to internal and external emotional stimuli need to be elucidated. The social and emotional responses of people with alexithymia are discussed and recommendations for future research are presented.

## 

Alexithymia, initially proposed by Sifneos (1972) [1], refers to impairment of the ability to identify and describe one's own feelings and emotions. This construct may be related to the impaired emotional regulation observed in a broad spectrum of psychosomatic phenomena (defined as bodily symptoms affected by psychosocial factors) and to psychiatric disorders associated with altered emotional processing. Although it is not a clinical diagnosis, alexithymia was initially proposed as an explanation of the clinical features seen in patients with psychosomatic disorders. It has been suggested that this personality trait might underlie the onset or exacerbations of physical symptoms.

Alexithymia is an important topic in the field of psychosomatic medicine [2]. It has been reported to have an effect on both the onset and/or progression of psychosomatic disorders. Neuroimaging of alexithymia is in its infancy, and there are not so many functional brain imaging studies which have attempted to clarify brain mechanisms related to alexithymia. A PubMed search identified 1,032 studies with the term ‘alexithymia’ in the title, but only 39 had used a neuroimaging technique, such as functional magnetic resonance imaging (fMRI), positron emission tomography (PET), single-photon emission computed tomography (SPECT), near-infrared spectroscopy (NIRS), or Event Related Potentials (ERP). Because experimental paradigms differ among studies, no single study can clarify the full picture of the neural basis of alexithymia; thus there is a need to evaluate the literature as a whole to determine patterns of key findings.

Several neuropsychological models of alexithymia have been proposed. MacLean [3] introduced the term “visceral brain” (= limbic/subcortical area) and suggested that interference with communication between the visceral brain and neocortical areas causes the lack of capability to identify and verbalize feelings. Instead, communication is in an “organic language,” which results in psychosomatic illnesses. Hoppe and Bogen [4] hypothesized that alexithymia is associated with an interhemispheric transfer deficit through the corpus callosum that reduces coordination and integration of the specialized activities of the two cerebral hemispheres. Some studies have emphasized a right hemisphere deficit in alexithymia [5,6] based on the hypothesis that right hemisphere plays a more important role in emotion processing than the left, which is engaged in language processes [7,8]. Dysfunction of the anterior cingulate cortex has been frequently argued, e.g., [9], and others have focused on neural substrates, such as the amygdala, insula, and orbitofrontal cortex (see the review in [10]). To date, there is no single conclusive hypothesis; possibly because these models interact with each other. No neuroimaging studies have been done that compare these various models of alexithymia.

Recent reviews focused on alexithymia and its neural associates are notable. Grynberg et al. [11] did a detailed review of neuroimaging studies on alexithymia and proposed interesting findings, but presented only findings from neuroimaging studies in which facial emotion was presented as stimulus. A recent review paper by Kano and Fukudo [12] focused on a variety of characteristics in alexithymia and, importantly, they pointed out stronger activation on the physiological and motor-expressive levels and less activation in the cognitive-experiential domains of people with alexithymia, with a limitation that the review was basically of their own studies. Wingbermühle et al., [10] did a nice overview of the theories proposed for alexithymia, its research, and the status of neuroimaging studies in which they listed the brain regions regarded as important. Although we accept the hypothesis that certain cognitive functions are ‘localized’ in specific brain region, recent studies in neuroscience show the complexities of processing by the brain and indicate that most brain regions are ‘multifunctional’, e.g., a region of a person may be activated in response to task A, but be deactivated in response to B, which suggests that the region is not engaged in a specific mental process. Therefore, when reviewing functional neuroimaging studies it is very important to consider the ‘context’ of the study, the paradigm and design. For instance, a conclusion can not be made that a person with alexithymia has hypo-(or hyper-) function in some specific brain region only by focusing on the localization of the activated/deactivated regions in an alexithymia study. Thus what is needed is the categoraziation of detailed ‘contexts’ of the tasks adopted in neuroimaging studies, which would provide insights into the functionality of a specific mental process or its disturbance, such as alexithymia.

We focus on certain core aspects of alexithymia and have attempted to identify brain regions and networks associated with alexithymia. Moreover, we suggest future research directions based on current knowledge. A review of the experimental paradigms used in prior studies indicated that they could be divided into four categories: 1) External emotional stimuli; 2) Imagery and fantasy; 3) Somatosensory or sensorimotor stimuli; and 4) Stimuli containing a social context. Findings relevant to each of these categories are separately discussed.

## External emotional stimuli

A plausible and simple hypothesis about alexithymia is that the condition is characterized by disturbed emotional processing. Researchers initially attempted to identify whether or not people with alexithymia showed different neural responses to external emotional triggers, such as pictures of facial expressions or to emotional situations.

**Figure 1:**
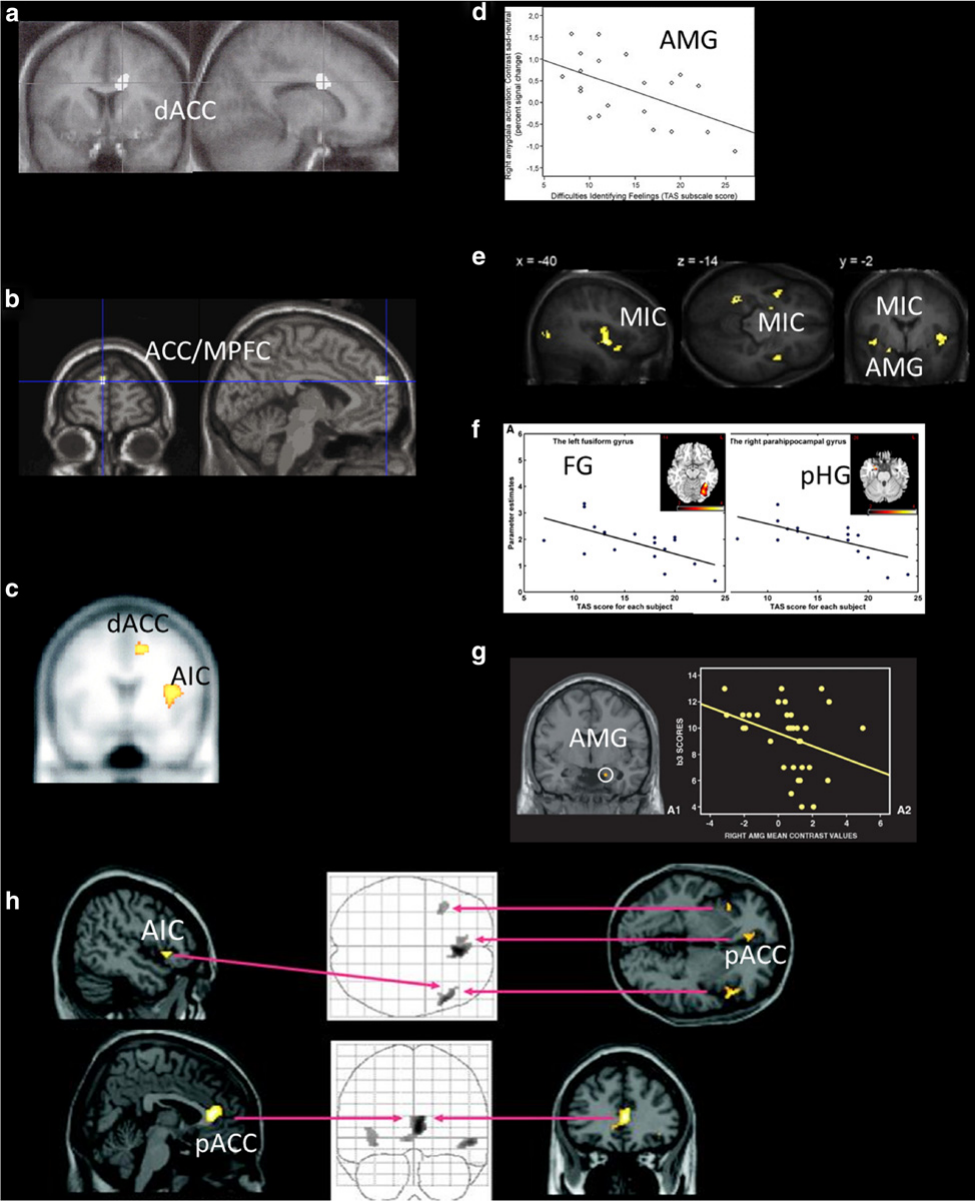
**Reduced brain response in alexithymia relative to external** (**visual**) **affective stimuli.** The plot figures show correlation between neural responses and alexithymia scores.dACC; dorsal anterior cingulate cortex, AIC; anterior insula cortex, MPFC; medial prefrontal cortex, AMG; amygdala, FG; fusiform gyrus, pHG; parahippocumpal gyrus, MIC; mid insula cortex, pACC; pregenual anterior cingulate cortex.Images are reproduced, with permission, from Ref. [9] for (**a**) ©, Ref. [14] for (**b**), Ref. [19] for (**c**), Ref. [20] for (**d**), Ref. [21] for (**e**), Ref. [22] for (**f**), Ref. [23] for (**g**), Ref. [24] for (**h**).

A pioneering study using H_2_^15^O-PET [9] showed that neural responses in the anterior cingulate cortex (ACC), also known as Brodmann's area 24 (BA24), to emotional films were positively correlated with individual scores on the Levels of Emotional Awareness Scale (LEAS) [13] (Figure 1a). The ACC is the region involved in attention and response selection. This result suggests that the ACC plays a critical role in emotional awareness, a characteristic deficient in people with alexithymia. Another study [14], using the International Affective Picture System (IAPS) and fMRI, reported that people with high alexithymia scores exhibit a low response in the medial prefrontal cortex (mPFC) and anterior cingulate cortex (ACC) (see Figure 1b). In this study, alexithymia was measured using the Toronto Alexithymia Scale (TAS-20) [15-18] and evocative pictures were used as emotional stimuli. The TAS-20 is a self-administrated questionnaire that was developed to measure individual alexithymia tendencies and that focuses on 1) difficulty in identifying feelings, 2) difficulty in describing feelings, and 3) externally oriented thinking. In another study, people with alexithymia showed reduced activation, as measured by H_2_^15^O-PET, in the dorsal ACC and right anterior insula (AI), especially in response to pictures depicting angry facial expressions [19] (see Figure 1c). These findings consistently showed that people with the traits of alexithymia exhibit reduced response to external (i.e., visual) stimuli, which is associated with hypoactivity in the ACC/mPFC, and AI.

The anterior cingulate, medial prefrontal, and insula areas form a network that is thought to work cooperatively in tasks associated with self-recognition (see [25,26]). Large spindle neurons (i.e., von Economo neurons (VENs) [27]) are distributed densely in this network, particularly in complex organisms, such as humans. Thus, the ACC-AI network is considered to be important for social development. For instance, patients with front-temporal dementia (FTD), which is characterized by the atrophy of areas including the ACC-AI network, have severe disturbances in self-recognition and social communication. The results of neuroimaging studies suggest that people with high alexithymia scores exhibit impaired recognition of their own emotional states due to a dysfunction of the ACC-AI network, given these regions' important role in self-awareness.

Other neuroimaging investigations of alexithymia have identified altered neural states in other affective brain areas. For example, one study focused on amygdala activation [20] showed that people with higher alexithymia scores had low amygdala response to subliminal (sad) facial pictures presented using a backward-masking technique (an emotional face is presented very briefly (~30 ms) followed by a supraliminal neutral face, so that subjects are unaware of the presence of the emotional pictures) (see Figure 1d). The same research team [28], using similar methods, i.e., subliminal (‘masked’) facial expressions, demonstrated a lower response in the fusiform area, which is known to be important for facial recognition, in people with high difficulty in identifying emotions (DIE) subscale scores on the TAS-20. A similar study using masked, sad facial expressions showed that people with high alexithymia scores had lowered neural responses in the amygdala, insula, superior temporal area, occipito-parietal area, and parahippocampus [21] (see Figure 1e). Masked ‘surprised’ faces also activated the fusiform and parahippocampal areas, and higher individual TAS-DIE scores were associated with lower activation in these regions [22] (see Figure 1f). Amygdala response to observed gestures expressing ‘fear’ is low in people with high TAS-DIE scores (see Figure 1g). These studies show that people with alexithymia exhibit reduced activation in the fusiform region, the parahippocampal gryi, and the amygdala, which are engaged in the visual processing of affective stimuli from the outer world. Another study using angry affective facial stimuli showed reduced right caudate activation in people with high alexithymia scores [29]. Neural activity in the amygdala, posterior cingulate cortex (PCC), and anterior cingulate cortex (ACC) was negatively correlated with the level of alexithymia, even in anorexia nervosa patients [30]. Another neuroimaging study focused on post-traumatic stress disorder (PTSD), in which the traumatic event was an automobile accident [24]. During fMRI scanning, patients with PTSD performed tasks that were designed to remind them of a traumatic event of their own. Patients also completed the TAS-20. The results indicated that people with high alexithymia scores showed low activation in areas that are important for processing affective information, specifically the anterior insula and pregenual ACC (see Figure 1h). Note that one study that used affective pictures and facial expressions showed exceptionally different results [31]. People with alexithymia exhibited increased dorsal-supragenual ACC activity. In this study, however, neural response to affective pictures was not contrasted with the response to neutral pictures, Thus, the task might not illuminate purely emotional component, which would account for the inconsistency with our findings. The reasons for this inconsistency are not clear at present.

In summary, alexithymia has been shown to be associated with reduced affective brain processes in response to external emotional triggers. This conclusion coincides with the empirical impression of clinicians that people with alexithymia look emotionally dull and unaffected [1,32-34].

### Imagery and fantasy

Another important factor associated with alexithymia is the limited ability to fantasize and incorporate imagery. This concept was originally included in the factors measured by the TAS, as one of its subscales, but later eliminated, because the validation of the ‘fantasy’ factor was not successfully established as an independent factor. Although limited fantasy and imagery have been regarded as important factors, few studies have been done on the role of imagery and fantasy in alexithymia, possibly because this factor is not currently included in the TAS-20.

**Figure 2:**
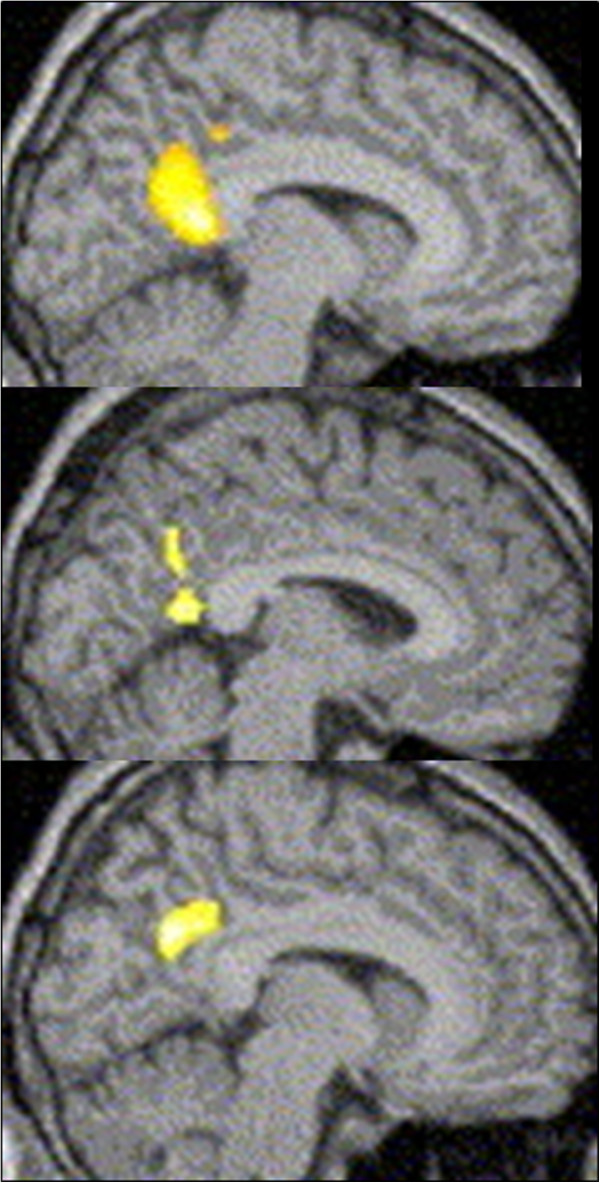
**Reduced brain activation in the posterior cingulate cortex of people with alexithymia during an imagery task.** Upper: Future happy imagery condition, Middle: Past happy imagery condition, Lower: Future happy imagery condition. Images are reproduced, with permission, from Ref. [35] ©.

In one neuroimaging study focusing on imagery ability [35], participants identified as alexithymic by TAS were required to imagine ‘possible future happy things’ or ‘past happy things,’ among others, during fMRI scanning. The results showed that the participants with high alexithymia scores showed reduced activation in the posterior cingulate cortex relative to participants who scored low in alexithymia. These results are summarized in Figure 2 and indicate that people with alexithymia may have reduced self-originating brain function, such as the accumulation of internal visual images.

### Somatosensory or sensorimotor level stimulus

As described previously, neuroimaging studies have shown that neural responses to external emotional triggers and internal imagery are abridged in alexithymia. Interestingly, very different results were obtained when the task paradigm included a context closely associated with something ‘physical” rather than emotional. That is, participants showed mostly *enhanced* brain activity in somatosensory or sensorimotor areas in response to such tasks.

**Figure 3:**
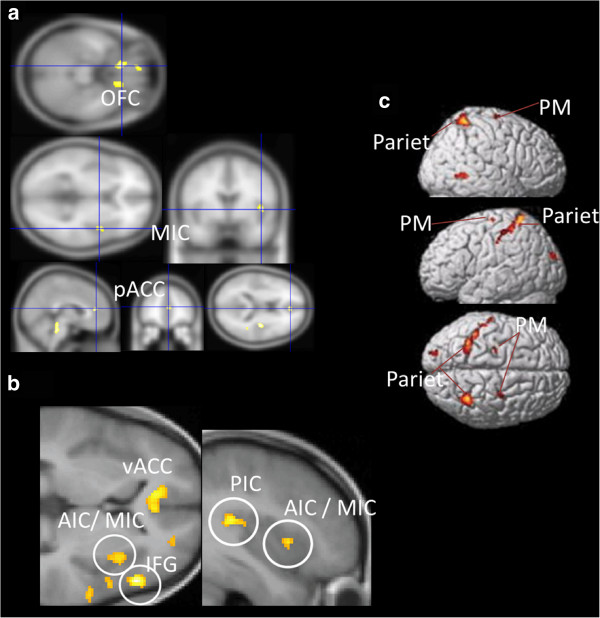
**Enhanced brain activation in response to somatosensory or sensorimotor stimuli.** OFC; orbitofrontal cortex, pACC; pregenual anterior cingulate cortex, vACC; ventral anterior cingulate cortex, AIC; anterior insula cortex, MIC; mid insula cortex, PIC; posterior insula cortex, IFG; inferior frontal gyrus, Pariet; Parietal cortex, PM; Premotor cortex. Images are reproduced, with permission, from Ref. [36] for (**a**) ©, Ref. [37] for (**b**), Ref. [38] for (**c**).

In one neuroimaging study using H_2_^15^O-PET [36], a balloon inflator was inserted into the colon of each participant, and cerebral blood flow was measured during interoceptive stimulation of the colon by inflating the balloon. The researchers found that people with higher alexithymia scores on the the TAS-20 exhibited high activation in the areas that are important for processing somatosensory information, including the ACC, right insula, midbrain, and orbitofrontal cortex, among others (see Figure 3a). Moreover, higher subjective scores were reported for the physical symptoms caused by the inflation, such as pain, feelings of stress, anxiety, and the urge to defecate. People with high alexithymia scores appear to be sensitive to both somatosensory stimulation, as indicated by their brain function, and to subjective perceptions.

In a study done in our laboratory [37], while being scanned by fMRI the participants observed pictures depicting hands and feet receiving painful stimulation, without experiencing any actual somatosensory stimulation. Although there was no actual pain stimulation, brain activation was observed in a ‘pain matrix,’ reflecting activation of the somatosensory cortex, insula, and ACC, as if the participants had actually received pain stimulation. The results indicated that, in comparison to the low alexithymia group, the high alexithymia group exhibited higher activation in brain areas associated with the affective pain matrix, such as the anterior and posterior insula, ventral ACC, and inferior frontal gyrus (see Figure 3b). Additionally, reduced activation in cognitive and executive areas, such as the dorsolateral prefrontal cortex, was also identified. Our team reported another neuroimaging study of alexithymia that was focused on the processing of physical information [38]. In that study, participants were placed in the scanner and they observed video clips illustrating object-related, goal-directed hand movements that involved reaching to grasp an object. The vedeo clips had been developed to reveal the "human mirror neuron system" (hMNS [39]; see more detail about hMNS in [40-43]). In doing this observation task, motor-related areas (premotor and parietal) are activated even though participants do not move their own hands. In this way, the task evokes sensorimotor level processing, such as automatic matching or a simulation system (i.e., hMNS), of the observation of an action and the internal model of the action. In this task, participants with higher alexithymia scores showed increased activation of the sensorimotor areas (premotor and parietal cortices) (Figure 3c).

Another neuroimaging study demonstrated that participants with high alexithymia scores exhibited higher activation of sensory and motor areas when they viewed emotional films [44], which is consistent with the findings of the studies described above.

Overall, these studies indicate that people with high alexithymia scores have enhanced primitive somatosensory- and sensorimotor-level brain function, which may contribute to amplifying physical sensations, such as pain. The results of neuroimaging studies are in line with clinical findings indicating that people with alexithymia tend to complain about external physical symptoms rather than reporting internal mental states.

### Stimuli containing social context

**Figure 4:**
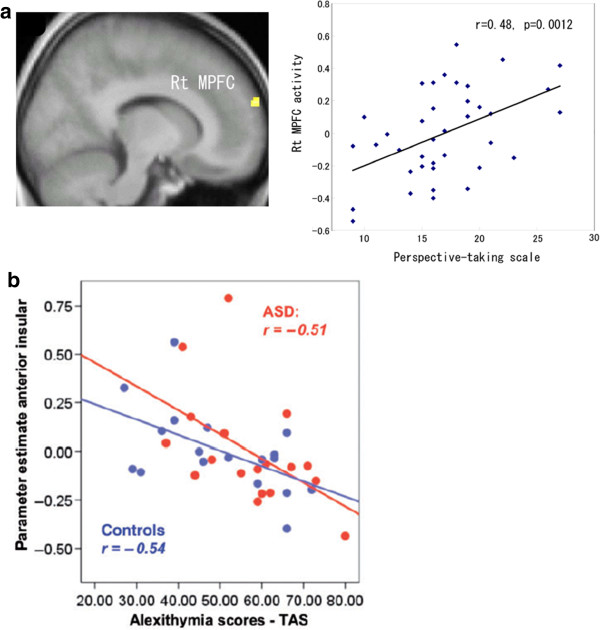
**Reduced brain activation during social tasks by people with alexithymia.** Mentalizing task and alexithymia. (Left) Decreased activity during the task. Rt MPFC; right medial prefrontal cortex. (Right) Correlation between MPFC activity and perspective taking scores. **a**) Correlation between alexithymia scores and activation in the anterior insula. Red: Autistic spectrum disorders (ASD), Blue: controls. Images are reproduced, with permission, from Ref. [50] for (**a**) ©, Ref. [52] for (**b**).

The original concept of alexithymia was defined as difficulty in being aware of and describing *one*’*s own* emotions, which leads to disturbance of emotional processes in the mind. Through our empirical and clinical experience, we have become aware that alexithymia is characterized by problems in social communication with *others*. Factors of autistic spectrum disorders, in which the central problem is a disturbance of social communication and the understanding of the minds of others, have been highly correlated with alexithymia traits [45-48]. The formation of alexithymia is likely to be related to some aspects of the developmental process [49], therefore, the development of self-recognition and the understanding of others may be inextricably linked. Alexithymia has also been shown to result in problems of understanding and expressing the mental states of others. We did a neuroimaging study of alexithymia [50] that focused on the ability to understand the minds of others (mentalizing or ‘theory of mind’). An animation task was used that required the participants to estimate the mental states of triangles moving like humans [51]. People with alexithymia had low mentalizing scores in response to this task. Next, fMRI was used to image the participants while they performed the task. The results showed that people with high levels of alexithymia exhibited low activation of the medial prefrontal cortex (mPFC; see Figure 4a), a central region for representing the mental state of the self and others. Although the concept of alexithymia as initially proposed was disturbance in the understanding of one’s own mind, it is noteworthy that people with alexithymia also have impaired understanding of mental states of others. Furthermore, mPFC activity has been correlated with ability in ‘perspective taking’ (i.e., the ability to take different perspectives from one’s own), as measured by a questionnaire (see Figure 4b), which suggests that the mPFC is involved in representing a common component in the understanding of the minds of the self and others. Moreover, the results indicated that the two different processes, the awareness of one’s own mental states and taking a perspective apart from one’s own (i.e., objectivizing oneself) are closely connected.

The final study we reviewed focused on the alexithymia of patients with autistic spectrum disorders (ASD) [52]. The participants were assessed with fMRI while they were given the information that a ‘person close to you is now receiving painful stimulation.’ In response, patients with high alexithymia scores showed decreased activity in the left insula, which is involved in estimating the pain of others and empathetic ability. This negative correlation between alexithymia and insula activity was constant across ASD and control groups, suggesting that alexithymia was more strongly associated than ASD with activity in the insula (see Figure 4c).

Emotional awareness may be associated with empathetic and imaginary functioning, and as a result of this relationship, alexithymia may include dysfunction in the social brain as well.

## Discussion

Neuroimaging studies of alexithymia were reviewed and the conclusions are summarized below.

1) People with alexithymia show reduced neural response in the limbic and paralimbic systems (e.g., amygdala, insula, ACC) to external affective (i.e., visual) stimuli. This suggests that affective arousal to external stimuli is disturbed in alexithymia.

2) In addition, people with alexithymia showed reduced neural response in the posterior cingulate cortex during an imagery task, which suggests that voluntary cognitive functioning, such as creating an image inside one’s mind spontaneously (not triggered by external events), is also disturbed in people with alexithymia.

3) In contrast, individuals with higher alexithymia scores exhibit increased neural response to stimuli accompanied by a ‘physical’ context, such as somatosensory or sensorimotor processes. This ‘hypersensitivity to physical level sensations’ may be associated with the fact that some patients (psychosomatic patients in particular) have a tendency to rely on, or to amplify their physical symptoms.

4) People with alexithymia show reduced activation in the mPFC, or insula, when they are engaged in cognitive processes, such as social tasks requiring mentalizing ability, or theory of mind. This may be indicative of an overlap between alexithymia and other psychiatric disorders characterized by impaired empathy, such as autistic spectrum disorders.

Overall, neuroimaging studies indicate that people scoring high on alexithymia measures exhibit either dullness to external affective triggers or hypersensitivity to internal and direct physical sensations, or both. Together with their reduced cognitive ability, such as mentalization or imagery ability, these features of alexithymia might clinically manifest as the inability of a person to express their own emotions and/or a tendency to become dependent on physical complaints. This reminds us of MacLean’s theory that cognitive processes in neocortical areas do not reach the visceral brain. We did not examine the lateralization problem (right hemisphere and inter-hemisphere dysfunction), but it will be important to address it in the future studies.

The findings seem to very well fit the theoretical construct of emotion proposed by Lane & Schwartz [53] in which emotional awareness can be graded in different ‘levels’ based on the cognitive-developmental theory of Piaget [54]. In this model, awareness of physiological cues and awareness of action tendencies are graded in the lower level. These types of fundamental awareness are the basis of higher cognitive levels of emotional awareness, such as differentiating emotions, even among different persons in social settings. The theory seems consistent with the findings of neuroimaging studies that people with alexithymia rely on a lower level of emotional awareness (i.e., physical/action level) and that their higher cognitive awareness is rather compromised. People with alexithymia may be ‘stagnating’ at the lower levels of emotional awareness.

An example of the clinical phenotypes that shows a primitive form of affective experience is panic disorder; (see the discussion in [55]). Individuals with panic disorder cannot symbolize and regulate states of emotional arousal and are overwhelmed by a host of bodily sensations and the fear of losing control. Their mind is focused on somatic sensations, and internal arousal is expressed directly as generalized autonomic discharge via somatic pathways without any modification by higher-order cognitive processes. Several studies have suggested that panic anxiety is due to an alexithymic deficit in cognitive processes of lower level affect, e.g., [56]. Such a feature of panic attack is quite consistent with the idea that people with alexithymia are focused on the somatosensory level of emotional awareness. There are no neuroimaging studies of panic disorder that focus on alexithymic features such as somatosensory tendencies. Such studies would be of great interest.

Other studies suggest that people with alexithymia are low on interoceptive awareness, e.g., [57], which might translate into the idea that people with alexithymia are not sensitive to their own physical sensations. This idea seems to conflict with the results of recent neuroimaging studies that showed that alexithymia is associated with hypersensitivity to internal and direct physical sensations. One caveat here is about the way that a tendency toward alexithymia is measured and reported in neuroimaging studies. The construct of alexithymia has been operationalized differently in different studies. In most studies, however, a self-administrated questionnaire, such as the TAS-20, has been used as the golden standard of alexithymia measurement. Considering the definition of alexithymia, however, people with alexithymia often have difficulty with self-awareness and difficulty looking into their own minds, and would be difficult for them to estimate correctly their own abilities in emotional awareness when answering the questionnaire. Perhaps some of the people who score high on alexithymia in the TAS are simply ‘oversensitive’ to themselves or highly ‘somatized’ (e.g., have a tendency to enhance somatic symptoms even without any organic physical change). This might lead to the results of neuroimaging studies that show physical hypersensitivity for people with “alexithymia” measured by self-reported questionnaires. Another problem about self-reported measures is that a person’s “criteria” for judging their own ability would greatly influence the score of a questionnaire that requires reflective estimation of that person’s own ability. Self-reported scores for alexithymia would be elevated even if the subject is merely ‘strict’ about his/her self-estimate. One solution is adopting an observer-based measure for use with a self-reported measure.

Bermond and Vorst proposed the inclusion of affective and cognitive facets to measure alexithymia [58,59]. They hypothesized that alexithymia is divided into two subtypes. Type I alexithymia is characterized by the constriction of both the emotional experience and the cognition accompanying the emotion, and they manifest a decreased physiological arousal. On the other hand, Type II alexithymia is defined by a selective deficit of cognitive facets (identifying, analyzing and verbalizing emotions) accompanied by an intact emotional experience and a normal physiological arousal. In validating their questionnaire (BVAQ) they found that the TAS-20 measures are only correlated with the cognitive facets of BVAQ, i.e., TAS-20 would identify people with either Type I or II alexithymia who have cognitive deficits but do not necessarily show impoverished emotional reactivity. It is possible that people who score high on TAS-20 would show relatively high response to interoceptive/physical triggers if they are categorized as Type I, but relatively low affective response to external triggers if categorized as Type II. The biggest question here is if these characteristics –dullness to external affective triggers and hypersensitivity to physical triggers – *coexist* in a single person or if they should be categorized into the different subtypes. Another question is if alexithymia is caused by combination of co-existing deficits or a single neurocognitive deficit sufficient to produce similar alexithymic characteristics. Future studies administering different paradigms in the same sample could bring us answers to these questions.

In fact, TAS-20 scores have shown high correlation with neuroticism scores [49,60], as well as with somatization measured by questionnaires or interviews [61-64]. Alexithymia identified only by a self-administrated questionnaire might be better explained as ‘physical neuroticism,’ in which functional symptoms are amplified through rumination focused on oneself. This idea reminds us of the hypothesis that people with panic disorder who have high TAS scores are focused on and overwhelmed by their own physical sensations. The only difference between neuroticism and alexithymia in neuroimaging studies is in the reactivity to emotional triggers from the outside world; while people with alexithymia measured by questionnaires show mostly reduced activation (such that they are ‘cold blooded’), people with high neuroticism consistently show hyper-activation in response to similar tasks, especially in the amygdala [65-68]. Questionnaires for alexithymia might, in a sense, contribute to “successfully” selecting their specific focus on their own physical sensation.

Thus, a provocative question here is whether or not we can call this physical neuroticism ‘alexithymia’ [57]. As Sifneos emphasized at every turn, what is really needed for physicians is to learn to recognize the difference between neurotic and alexithymic patients [69]. The answer would be negative if we accept that the core concept of alexithymia is cognitive deficits in emotional awareness (i.e., ‘no words for feeling’, or ‘difficulty in identifying and expressing emotions’). If we expanded the original definition of alexithymia, we would have accept that this physical tendency is included in alexithymia. This would simplify the matter: It depends on the definition of alexithyimia (see Table 1). Whether or not the self-reported questionnaires, like TAS-20, have successfully identified people with alexithymia in previous studies needs to be carefully examined. It is important to accept the fact, from neuroimaging studies, that some people who had high ‘alexithymia’ scores exhibit a way of emotional processing that relies specifically on their own physical sensations. It is highly possible that this tendency toward lower-level emotional processing promotes the aggravation of physical complaints, such as panic attack, somatized pain, and other functional physical sensations.

**Table 1:** Future tasks for neuroimaging studies of alexithymia

	
•	Problems of the definition of alexithymia
✧	What is alexithymia and what is not alexithymia? Is emotional numbing in PTSD included in alexithymia? How about ‘depersonalization’ or ‘repressive coping style’? etc.
✧	Is it acceptable that alexithymia includes negative affective components like depression, anxiety, neuroticism, high distress etc.?
•	Clinical aspects of alexithymia
✧	Alexithymia originates from the characteristics of psychosomatic patients, but normal samples were mainly selected for past neuroimaging studies of alexithymia. How do we interpret the clinical meaning of the results for alexithymia in contrast to healthy samples?
✧	What are the common components and differences between healthy individuals with high TAS-20 scores and patients with somatic symptoms?

If we accept the use of self-administrated questionnaires, we must be sensitive to which factors in the questionnaire are used. For example, TAS-20 has three subscales; difficulty in identifying feelings (DIF), difficulty in describing feelings (DDF), and externally oriented thinking (EOT), but the differences in the three factors have not been investigated thoroughly in neuroimaging studies. Although the three factors are used as components for identifying the same personality traits related to alexithymia and heavily overlap each other, it has been reported that EOT is somewhat different from the other two. DDI and DDF are considered to be ‘affective’ facets of alexithymia, but EOT is less correlated with the other two [16,70] and is thought to reflect more the ‘cognitive’ dimension of alexithymia [71,72]. Among the three TAS subscales, EOT is the only factor that has a significant (negative) correlation with the levels of emotional awareness scale (LEAS) [73,74]. It is quite noteworthy that EOT does not correlate with depression but correlates negatively with the degree of interoceptive awareness, which is scaled as accuracy in a heartbeat detection task [57]. Emotional components are correlated with depression, but EOT is not [75,76]. An EOT style directs thought at external reality from internal focus or attention on feelings [77,78]. EOT is specifically associated with paying less attention to one's emotions and is correlated with attention to emotional information as measured with an emotional Stroop task [77]. These two facets (emotional and cognitive/attentional) might best be considered separately when TAS-20 is used in neuroimaging studies.

**Figure 5:**
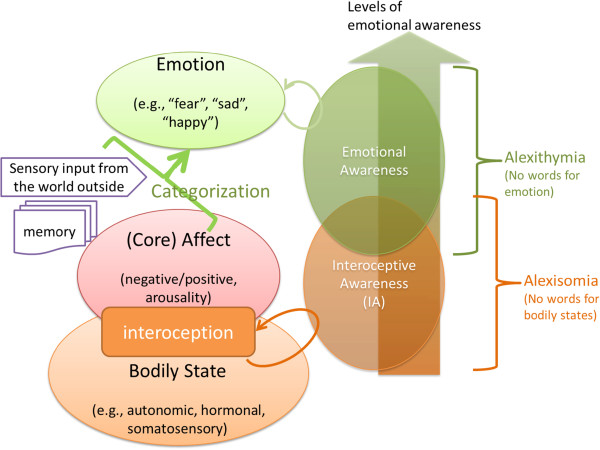
**Neuropsychological model of emotional awareness and possible mechanism of alexithymia and alexisomia.** Bodily states including autonomic, hormonal, and somatosensory status are the basis of the organism’s basic affective states, called ‘Core Affect’ [80,82]. This is hypothesized as variable states of brain and body, consisting of the intensity (arousal level) with negative or positive affective value (i.e., valence). The Core Affect is mainly formulated from the information from the body known as ‘interoception’. Our information available for constructing our mental states should be 1) the Core Affect, i.e., information from the body, 2) information from the past stored in one’s brain, called ‘memory’, and 3) information from the world outside the body (e.g., visual, or auditory input etc.). These three information sources are ‘categorized’ in the brain and a certain mental state is formulated (which might be a “thought”, a “feeling”, or an “emotion” at the time); see [83,84]. Emotional awareness is assumed to have “levels“ [13]. At the lower level of emotional awareness, the target of awareness is the core affect or basic level of affective states, which is strongly connected to physiological or bodily status, or interoceptive awareness. We can postulate that ‘alexisomia’ involves difficulty in the lower levels of emotional awareness, i.e., interoceptive awareness or awareness of core affective states. On the other hand, higher levels of (more cognitive) emotional awareness should include a ‘categorization’ process that integrates the three sources of information and constructs a mental state that is ‘experienced’. The core of the mechanism of ‘alexithymia’ could be a problem in categorization or cognitive awareness or metacognition of categorized emotional states (e.g., can’t identify or express one’s own emotional state as ‘angry’). If we accept that interoceptive awareness is fundamental to the construction of the emotional experience and awareness, alexithymia (difficulty in emotional awareness) and alexisomia (difficulty in interoceptive awareness) are closely connected to each other.

Finally, an intriguing alternative concept, called ‘alexisomia,’ has been coined, referring to difficulty in the awareness of somatic sensations in addition to a lack of awareness of emotions. This construct was proposed by Ikemi over 20 years ago [79] – and it might be an important variable in the pathology of psychosomatic disorders. ‘Core affect’ is a psychological term that indicates states experienced as simply feeling ‘good or bad’ or ‘energized or enervated’ in response to emotional experiences, mood, or any other emotionally-charged event [80]. This core affect is a basis of every emotional experience and is closely connected to a person’s physical status, such as the autonomic arousal level. William James [81] proposed the idea that emotions are often accompanied by bodily responses and that we can sense what is going on inside our body by use of interoceptive awareness. He claimed that different emotions feel different from one another because they are accompanied by different bodily responses and sensations. If one's awareness of bodily states is the basis of emotional awareness, there should be a problem of awareness of bodily sensations (i.e., alexisomia) or deficits of emotional awareness underlying alexithymia (see Figure 5 and the discussion in the legend). Thus, previous neuroimaging studies based on sampling by questionnaires did not adequately address this important problem, the lack of awareness of physical sensations, or alexisomia. Observer-based measurements such as interviews are thus necessary. Alexisomia should be addressed as an important facet of alexithymia in future neuroimaging studies.

## Competing interests

The authors declare that they have no competing interests.

## Authors’ contributions

YM and GK collected the papers referenced. YM drafted the paper and GK edited it. Both authors read, discussed, and approved the final manuscript.
